# An unsupervised classification method for inferring original case locations from low-resolution disease maps

**DOI:** 10.1186/1476-072X-5-56

**Published:** 2006-12-08

**Authors:** John S Brownstein, Christopher A Cassa, Isaac S Kohane, Kenneth D Mandl

**Affiliations:** 1 Children's Hospital Informatics Program at the Harvard-MIT Division of Health Sciences and Technology, 1 Autumn St, Boston, MA, USA; 2 Division of Emergency Medicine, 300 Longwood Ave, Children's Hospital Boston, Boston, MA, USA; 3 Department of Pediatrics, 300 Longwood Ave, Harvard Medical School, Boston, MA, USA

## Abstract

**Background:**

Widespread availability of geographic information systems software has facilitated the use of disease mapping in academia, government and private sector. Maps that display the address of affected patients are often exchanged in public forums, and published in peer-reviewed journal articles. As previously reported, a search of figure legends in five major medical journals found 19 articles from 1994–2004 that identify over 19,000 patient addresses. In this report, a method is presented to evaluate whether patient privacy is being breached in the publication of low-resolution disease maps.

**Results:**

To demonstrate the effect, a hypothetical low-resolution map of geocoded patient addresses was created and the accuracy with which patient addresses can be resolved is described. Through georeferencing and unsupervised classification of the original image, the method precisely re-identified 26% (144/550) of the patient addresses from a presentation quality map and 79% (432/550) from a publication quality map. For the presentation quality map, 99.8% of the addresses were within 70 meters (approximately one city block length) of the predicted patient location, 51.6% of addresses were identified within five buildings, 70.7% within ten buildings and 93% within twenty buildings. For the publication quality map, all addresses were within 14 meters and 11 buildings of the predicted patient location.

**Conclusion:**

This study demonstrates that lowering the resolution of a map displaying geocoded patient addresses does not sufficiently protect patient addresses from re-identification. Guidelines to protect patient privacy, including those of medical journals, should reflect policies that ensure privacy protection when spatial data are displayed or published.

## Background

Geocoding patient data – translating the plaintext addresses of patients into longitudes and latitudes – has become routine and enables display and analysis of disease patterns. Many public health surveillance systems and academic investigations rely on specific case locations for identifying patterns, correlates, and predictors of disease [1-3]. Maps that display such geocoded health data are frequently presented publicly and published electronically and in print.

However, publishing patient address locations on maps also creates a risk of re-identification of individuals [4-7]. We recently reported an inadvertent breach of privacy across five major medical journals, identifying 19 articles from 1994–2004 that include maps with patient addresses plotted as individual dots or symbols [4,5]. From these publications, over 19,000 patient addresses are plotted on map figures. We demonstrated through a process of reverse identification that the home addresses of many of these patients could be discovered, despite the low resolution of the disease maps.

Here, we provide the details of that method. We rely on unsupervised classification of the spectral properties of the map image to identify case locations. Since we do not have available to us the original addresses of the patients represented in the published maps, we devised an indirect approach relying on simulation.

## Methods

**Figure 1:**
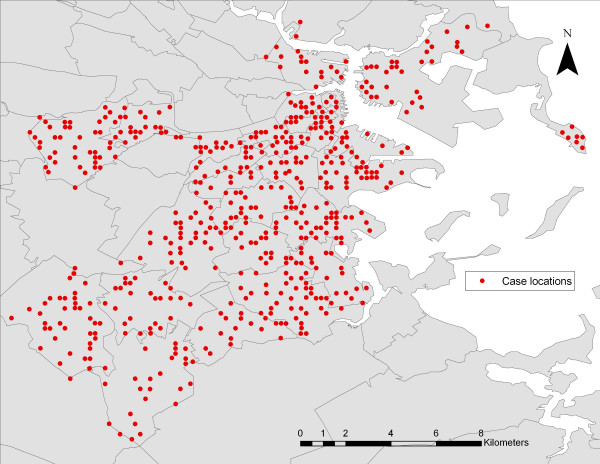
**Prototypical patient map for Boston, Massachusetts**. The image displays 550 addresses selected by stratified random sampling design. The original JPEG image used in the analysis had a resolution of 50 dots per inch (550 × 400 pixels), a file size of 129 kb and a scale of 1:100,000. This would be a typical output for web display and usually lower resolution than would be shown in a slide presentation or in a peer-reviewed publication.

We sought to quantify the degree of re-identifiability of patient home addresses from published maps. To accomplish this, a hypothetical low-resolution map of geocoded patient addresses is produced and then the accuracy with which patient addresses can be resolved (reversely identified) through a five step process is measured. First, an original, prototypical patient map for an urban metropolitan area in Boston, MA was produced (Figure 1). Using building parcel outlines for the city of Boston,[8] we generated a synthetic or hypothetical set of patient addresses by randomly selecting buildings. Cases were assigned by a stratified sampling design of building parcels to achieve a distribution representative of all building and population densities in the city. Buildings were selected with equal spacing of 0.02 degrees. A total of 550 addresses were randomly selected. Centers of the selected building were then calculated and plotted on a county map of Boston to represent patient addresses [9]. One important issue is that our use of the building footprint for geocoding does not mirror the reduced accuracy obtained from geocoding addresses. Address geocoding will have a series of associated errors that may be related to the underlying structure of a geographic area (e.g.: road length, parcel size, housing density)[10].

We created a JPEG image with a resolution of 50 dots per inch (dpi), 550 × 400 pixels, a file size of 129 kb and a scale of 1:100,000. This low resolution is typical for web display and is lower than generally used in slide presentations. Also the re-identification of patient addresses was evaluated using a higher-resolution map (266 dpi, 2926 × 2261 pixels, 712 kb, 1:100,000), often the minimum resolution for peer-reviewed publications.

There are several steps involved in reversely identifying a patient address. First, the sample map is scanned or imported into GIS software as an image file [11]. Second, the imported map is georeferenced. The cartographic projection of the map is used to set the coordinate system. Generally, the projection of a published map would be unknown and the correct projection would need to be found by manually matching the image of the map to an image of a correctly georegistered map of the same area. In this case, we have a priori knowledge of the map projection. In either case, ground control points are selected on the image using a corresponding vector outline of the map area to re-project the image file of patient locations and reference it to a coordinate system. In this example, an outline of counties around Boston provided by the US Census Bureau to set the ground control points [9]. The process of scanning and georeferencing the disease map parallels the methodology detailed by Curtis et al [7]. Third, using image analysis software [12], unsupervised classification of the georeferenced map is performed. Given the spectral properties of the image file, pixels are classified so that pixels representing the patient points are aggregated together. Fourth, a reclassified raster map (an image composed of individual pixel elements arranged in a grid) that only contains patient points is extracted and converted to a vector file. Finally, Coordinates of the patient points are then calculated.

Accuracy of reverse geocoding was measured as (a) the number of correctly identified patient addresses (b) the distance between the reversely identified address coordinate and the boundary of the building of the patient home address and (c) the number of buildings in which the patient could reside, given the reversely geocoded address. To calculate (c), we estimated the minimum buffer size from the predicted location needed to contain the centroid of the correct address. Accuracy in this case is therefore defined as the number of incorrect addresses within this buffer.

## Results

Our reverse identification method correctly identified 26% (144/550) of patient addresses precisely, from a sample map with low-resolution GIS output. We observed increased detection with the higher-resolution publication quality output to 79% (432/550) of patient addresses identified exactly.

**Figure 2:**
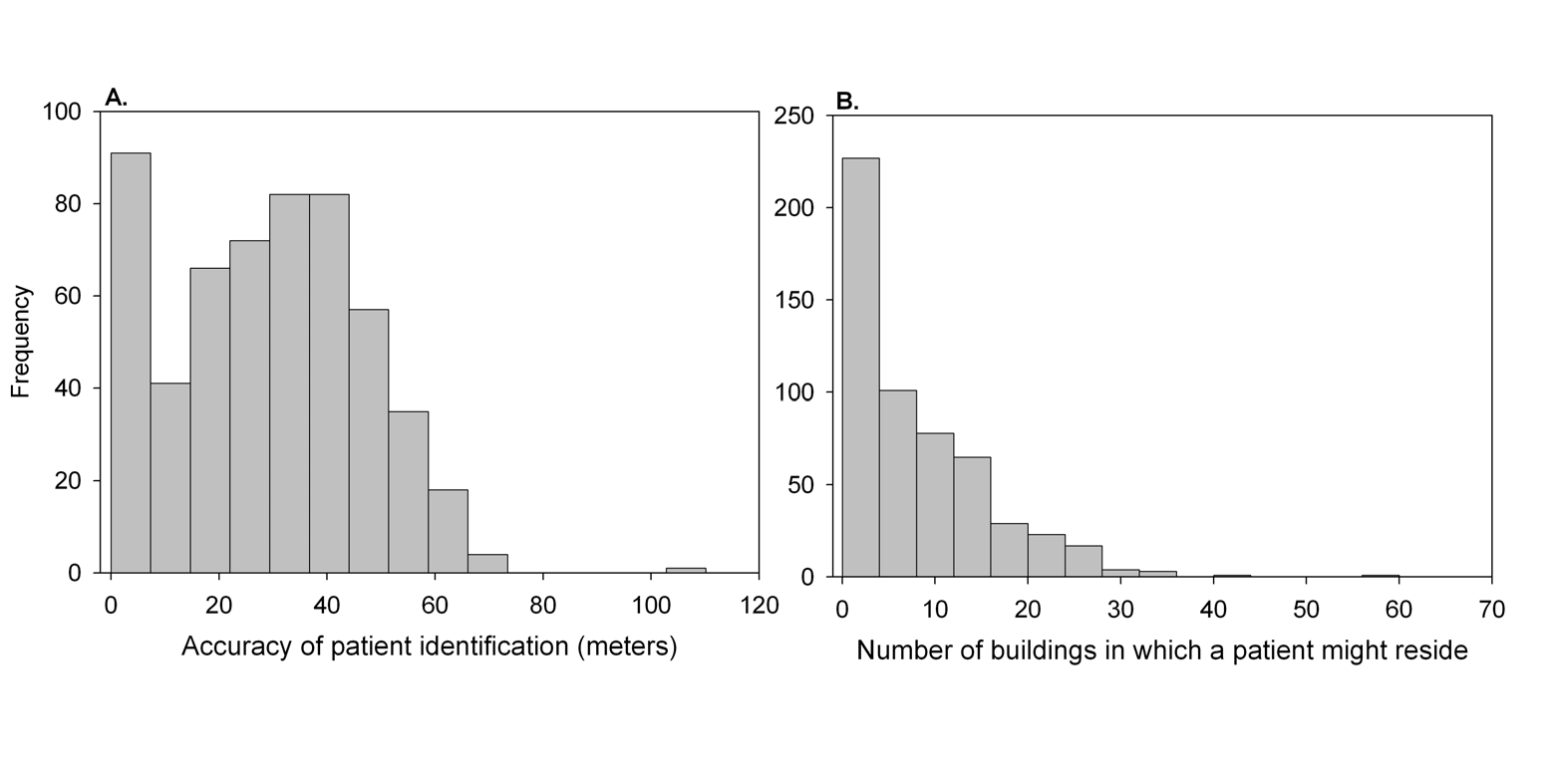
**Accuracy of reversely identifying patient location from a hypothetical low-resolution patient map in Boston, Massachusetts**. The accuracy of the reverse identification was determined by **(A) **the distance between the reversely identified and the original addresses and **(B) **the number of buildings in which the patient could reside, given the reversely geocoded address. The reversely geocoded location was on average within 28.9 meters (95% CI, 27.4–30.4) of the correct address. The mean number of buildings in which the patient might reside was 7.7 (95% CI, 7.0–8.3).

For the low resolution presentation quality map, reversely geocoded locations were on average within 28.9 meters (95% CI, 27.4–30.4) of the correct original address (Figure 2a). On average, correct patient address was identified within eight buildings (95% CI, 7.0–8.3). Overall, 51.6% of addresses were identified as being at any of five buildings, 70.7% at any of ten and 93% at any of 20 (Figure 2b). For the higher resolution publication quality map, all addresses were predicted within 14 m of the correct address. This distance is well within the footprint of most apartment buildings and even many single family residences. While most addresses (79%) could be identified to a single building, the maximum number of buildings in which the patient could reside, given the reversely geocoded addresses was 11 buildings.

**Figure 3:**
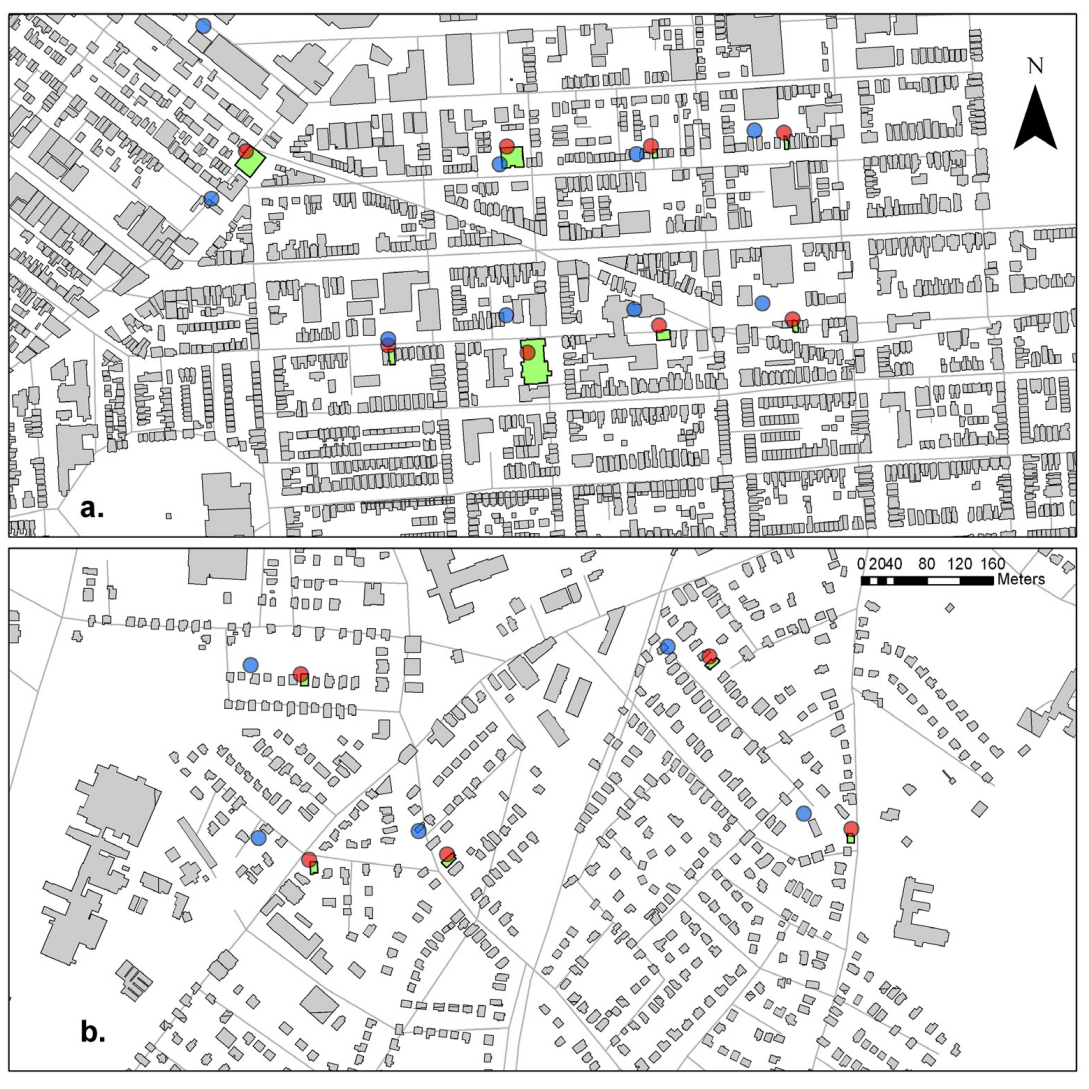
**Results of reversely identifying patient addresses in Boston, Massachusetts**. The green buildings are the randomly selected patient locations. The blue points are the predicted locations of the cases from the presentation quality map (50 dpi) and red points are predictions from the publication quality map (266 dpi). Proximities of the predicted to the actual location are displayed for both **(A) **a high density urban area and **(B) **a low density suburban area.

Predictions of patient location were accurate in both densely-populated urban settings as well as suburban regions, as illustrated in Figure 3. Among those addresses precisely identified, there was no observed effect of housing density on the rate of patient addresses re-identification. However, given the variation in number of individuals per housing unit, we expect that the anonymity of patients in suburban single family houses would be significantly reduced compared to urban areas. Locales with a high probability of living in large apartment buildings afford greater anonymity. In this study, we essentially controlled for the variability of geocoding accuracy by using building footprint data rather than address data. Previous research has shown that housing density may have substantial impact on address geocoding accuracy [10].

## Discussion

Our results demonstrate that even lowering the resolution of a map displaying geocoded patient addresses does not sufficiently protect patient addresses from re-identification. Despite the low quality of output sources, these images – based on high precision input sources – preserve positional accuracy. Using a low quality map that would serve the purpose of web or presentation display, we were able to precisely identify more than one quarter of all randomly selected home addresses and on average patients could be identified to a city block or within one of eight buildings. Using a map with minimum resolution for peer-reviewed publication, we could identify almost all patient addresses and on average patients could be identified within 14 m.

The ultimate accuracy of the patient re-identification will no doubt depend on the number of individuals residing at these addresses. In the case of multi-family apartment dwellings, address identification may still afford a certain level of privacy protection. In the case of single family dwellings, re-identification becomes much more likely. However, even in the best case scenario of an urban area multi-family apartment building, an additional concern is that individuals at these addresses can be fully re-identified when linked with other datasets or by using other characteristics supplied in the publication [13]. Previous research has shown that combinations of seemingly innocuous data is adequate to uniquely identify individuals with a high level of reliability [14]. For example, an experiment using 1990 U.S. Census summary data surprised the public health community by showing that datasets previously thought to be adequately de-identified, containing only 5-digit ZIP code, gender and date of birth, could be linked with other publicly available data (e.g., voting records) and used to uniquely identify 87% of the population of the United States [15]. Low-resolution maps of patient locations pose an additional risk to individual privacy – allowing considerably more precision in re-identification than might be expected. Although the Health Insurance Portability and Accountability Act Privacy Rule (Section 164.514) does not explicitly address the publication of such maps, certain formats of geographic data display most likely violate the spirit of that rule.

Curtis et al have also recently described a method to re-identify patients from published maps through manual outlining of case markers [7]. Though the vector-based approach of heads-up digitizing can be more accurate than raster-based unsupervised classification in certain circumstances, in this case, it may be difficult to find the true border of the case markers from a scanned paper-based maps (such as the newspaper article described by Curtis et al) or even low-resolution digital images. If the marker is not digitized accurately, then it follows that the centroid of this polygon will also less accurately reflect the original geocoded location. Our approach differs from the manual approach in that we rely on analyzing the spectral properties of the map image through unsupervised classification to automatically identify patient locations. The raster-based method based on the spectral properties of the image can provide a reliable means of re-creating the original vector file and systematically obtaining the center point of a low-resolution marker. This comparison, however, warrants further evaluation. Nonetheless, the results of the two papers are very similar in that they show that maps containing point data are vulnerable to patient address re-identification. These studies and our previous publication on this topic [5] should be viewed together informing policy around the display of geographic data.

The main question that should be asked by both authors and editors is what are the benefits and risks of point localization of patients? Is it necessary to publish maps of point locations, for the presentation of relevant results of research or are they presented merely for illustrative purposes? The answer to these questions should guide decisions on how to report disease maps [16]. If just for illustrative purposes, there are techniques available to visualize spatial data without revealing patient information [17]. For instance, a common approach to de-identifying such data has been to use ZIP or postal code rather than home address to protect anonymity. While usually appropriate for the reporting of study results, aggregation of data to an administrative unit poses constraints on the analysis and visualization of disease patterns [17-19]. Other approaches are available for masking geographic data, such as spatial masking of cases by randomly relocating cases within a given distance of their true location [20-23] or the population-density adjusted 2D Gaussian blurring approach which results in only a small reduction in sensitivity to detect clustering patterns [24]. These methods avoid these visualization constraints of data aggregation and afford sufficient privacy for publication without substantial loss to visual display. Masking methods provide more systematic and reliable means of de-identification rather than simply reducing map resolution. Spruill developed a measure of privacy protection for any mask, analogous to our measure of number of addresses within which the patient could reside [25]. Such a measure could be used by journal editors as a rule for not publishing maps of individual cases unless a certain value of anonymity was attained. This measure, often referred to as K-anonymity, could help to establish guidelines for the safe publication of disease maps [13,24].

Our approach relies on simulation, rather than attempting to re-identify patients from published maps. We chose this approach to avoid propagating any prior inadvertent disclosures of patient identity, and to avoid impugning particular authors or journals. An advantage of our approach is that since we know the value of the original plotted location, we can precisely measure the accuracy of re-identification. Our analysis also does not address the geocoding method. Accuracy of re-identification will also be dependent on the method for geocoding patient address. Use of a global positioning system (GPS) will provide greater accuracy then that of an address geocoder (automatic conversion from home address text to latitude and longitude using interpolation along street line data). When a geocoder is applied, the input data source will affect the accuracy of the estimate address coordinate. Many US-based studies rely on the freely available US Census TIGER line file as input to assign coordinates to addresses. Although TIGER line files differ in accuracy across the US, they rarely, if ever, approach the geometric accuracy of GPS coordinates or even more detailed commercial datasets. In fact, geocoding based on the free Census data available to most health researchers increases patient anonymity as the proportional placement of the address location can greatly affect geocoding accuracy [10,26]. Outside the US, street level data may not be available for address geocoding. Therefore, spatial analysis studies in these areas would rely on the more accurate GPS measures. By extension, greater positional accuracy is revealed in these studies. Our findings may therefore be highly pertinent for GIS-based studies in developing countries.

The issues we raise here have, of course, much wider implications than for just health data, including crime data, housing data (e.g.: Section 8 units, shelters for abused women, etc.), and other administrative data sets [20,27,28]. New spatial data standards that protect confidentiality while still effectively communicating information about spatial patterns require immediate evaluation [29].

## Conclusion

The publication of low-resolution disease maps poses an inherent jeopardy to patient privacy. Because the appropriate use of the patient address level data can bring real benefit to many areas of public health research that deal with spatial analysis, accidental disclosure of patient information from such maps may lead to constraints on obtaining geographically referenced health data. Thus, guidelines for the display or publication of health data are needed to guarantee privacy protection. Further, the editors of journals and textbooks should consider implementing policies to ensure the safe reporting of spatial data.

## Competing interests

The author(s) declare that they have no competing interests.

## Authors' contributions

JSB participated in the study concept and design, acquisition of data, analysis and interpretation of data, statistical analysis, drafting and revision of the manuscript. CAC and ISK participated in the study concept and design, and critical revision of manuscript. KDM participated in the study concept and design, analysis and interpretation of data, study supervision, and critical revision of manuscript. All authors have no conflicts of interest. All authors read and approved the final manuscript.
